# Non-invasive, Brain-controlled Functional Electrical Stimulation for Locomotion Rehabilitation in Individuals with Paraplegia

**DOI:** 10.1038/s41598-019-43041-9

**Published:** 2019-05-01

**Authors:** Aurelie Selfslagh, Solaiman Shokur, Debora S. F. Campos, Ana R. C. Donati, Sabrina Almeida, Seidi Y. Yamauti, Daniel B. Coelho, Mohamed Bouri, Miguel A. L. Nicolelis

**Affiliations:** 1 Neurorehabilitation Laboratory, Associação Alberto Santos Dumont para Apoio à Pesquisa (AASDAP), São Paulo, 05440-000 Brazil; 2 STI IMT, École Polytechnique Fédérale de Lausanne, Lausanne, Switzerland; 3 Associação de Assistência à Criança Deficiente (AACD), São Paulo, 04027-000 Brazil; 4 Biomedical Engineering, Federal University of ABC, São Bernardo do Campo, SP 09606-045 Brazil; 5 Department of Neurobiology, Duke University Medical Center, Durham, NC 27710 USA; 6 Duke Center for Neuroengineering, Duke University, Durham, NC 27710 USA; 7 Department of Biomedical Engineering, Duke University, Durham, NC 27708 USA; 8 Department of Neurology, Duke University, Durham, NC 27710 USA; 9 Department of Neurosurgery, Duke University, Durham, NC 27710 USA; 10 Department of Psychology and Neuroscience, Duke University, Durham, NC 27708 USA; 11 Edmond and Lily Safra International Institute of Neuroscience, Macaíba, Brazil

**Keywords:** Motor control, Sensorimotor processing

## Abstract

Spinal cord injury (SCI) impairs the flow of sensory and motor signals between the brain and the areas of the body located below the lesion level. Here, we describe a neurorehabilitation setup combining several approaches that were shown to have a positive effect in patients with SCI: gait training by means of non-invasive, surface functional electrical stimulation (sFES) of the lower-limbs, proprioceptive and tactile feedback, balance control through overground walking and cue-based decoding of cortical motor commands using a brain-machine interface (BMI). The central component of this new approach was the development of a novel muscle stimulation paradigm for step generation using 16 sFES channels taking all sub-phases of physiological gait into account. We also developed a new BMI protocol to identify left and right leg motor imagery that was used to trigger an sFES-generated step movement. Our system was tested and validated with two patients with chronic paraplegia. These patients were able to walk safely with 65–70% body weight support, accumulating a total of 4,580 steps with this setup. We observed cardiovascular improvements and less dependency on walking assistance, but also partial neurological recovery in both patients, with substantial rates of motor improvement for one of them.

## Introduction

Every year, about 250,000–500,000 people worldwide suffer a spinal cord injury (SCI) as a result of traffic accidents, falls, other traumatic accidents or violence^1^. SCI leads to severe impairments of the sensory, motor and autonomic functions below the lesion level, as well as secondary clinical conditions such as pressure ulcers, urinary tract infections, and osteoporosis. In addition to the gravity of these clinical effects, SCI incurs substantial financial costs, both for the individual and society. For patients diagnosed with the most severe cases of SCI (patients presenting no motor functions below the lesion (AIS B) and patients presenting neither motor nor sensory functions (AIS A)), at the chronic phase of the lesion (a year after the injury), the chances for a spontaneous recovery are negligible^2,3^. Up to now, there is no systematic approach to restoring neurological functions in such patients.

Over the past decades, a variety of new approaches for SCI rehabilitation have been introduced^4,5^. These efforts aimed at inducing activity-dependent plasticity^5^ by the utilization of robotic trainers^6–8^, epidural electrical stimulation^9–11^, body-weight support trainers^12^, brain-machine interfaces^11,13–15^, transcranial magnetic stimulation^16,17^ and surface functional electrical stimulation (sFES)^18,19^. Recently, our group has shown that a training protocol (the Walk Again NeuroRehabilitation protocol (WANR))^14,20^ – induced a significant level of neurological recovery in a group of eight subjects with chronic complete paraplegia (seven AIS A, one AIS B), by combining assisted walk training with robotic walkers, electroencephalography (EEG)-based brain-machine interface (BMI), and continuous visuotactile feedback. Extending our previous findings, the present study describes our group’s effort to further enhance clinical neurological recovery in severe cases of SCI by providing more selective lower-limb musculoskeletal recruitment through sFES, while maintaining our fundamental philosophy of activating both ascending and descending neural pathways during the neurorehabilitation process. As such, the BFNR (BMI, sFES, NeuroRehabilitation) protocol described here integrates four key elements to potentiate recovery in patients with SCI: muscle activation through sFES^21–23^ (see^24^ for a review), balance control through body weight support^25^, real-time decoding of motor command (BMI)^14,20^ and sensory feedback through a portable haptic device^26^.

In recent years, BMI-FES (systems that use BMI to detect patients’ voluntary motor commands to trigger their muscle contractions through an FES) have been extensively studied as rehabilitation tools (some researchers call it therapy or partial restoration) for patients with severe cases of stroke^27–29^, to potentiate recovery of upper-limb^28,30,31^ or lower-limb motor function^32^ (see^33^ for a review).

On the other hand, several studies with SCI patients have shown remarkable results when using BMI-sFES technology as an assistive device, by bypassing the spinal cord lesion and permitting the patient to perform motor functions^34,35^. However, to date, little is known about the potential of the BMI-sFES approach as a true rehabilitative tool/therapy for patients with SCI. To our knowledge, only one study with rats has rigorously tested this hypothesis: Bonizzato and colleagues^15^ recently showed that a training paradigm integrating BMI with epidural stimulation accelerated and enhanced the animal’s neurological recovery when compared to training that used only the epidural stimulation.

Here, we studied both the assistive and the restorative aspects of a novel BMI-sFES system with two patients with chronic SCI. First, we demonstrated the validity of each aspect of our approach: a custom 16 channel sFES system, a closed-loop proportional- integral (PI) controller to cope with muscle fatigue, the integration of a portable haptic device to cope with the lack of lower-limb sensory functions and a novel BMI protocol based on the detection of left and right leg motor imagery. Next, we tested our neurorehabilitation protocol with two patients with SCI (originally AIS A) who had been previously trained with the Walk Again Neurorehabilitation (WANR) protocol and, as a consequence, converted to AIS C^20^ (Supplementary Tables S1, S2). At the onset of the current protocol both patients presented motor functions more than three segments below the motor level (presence of knee extension (L3) for both patients); however, their overall motor score at that point in time was low (lower extremity motor score of 4 for P1 and 2 for P2 out of a maximum possible score of 50). To identify the effect of the BFNR protocol, we ran a series of neurological^36^, neurophysiological and functional (WISCI II)^37^ assessments at the onset and at the end of training. Overall, we observed a significant improvement in muscle responses and reduction of muscle fatigue in our two patients in response to the sFES. Patients also experienced an increase in muscle volume and functional improvement for walking (measured as the ability to walk 10 meters in full load, using only passive assistive devices, such as walkers, orthoses, and crutches). More remarkably, we also documented clinical neurological improvement in both patients. Indeed, one of these patients reached a substantial increase of 9 points for the lower extremity motor score (LEMS) after 22 sessions with the BFNR training.

## Results

**Figure 1:**
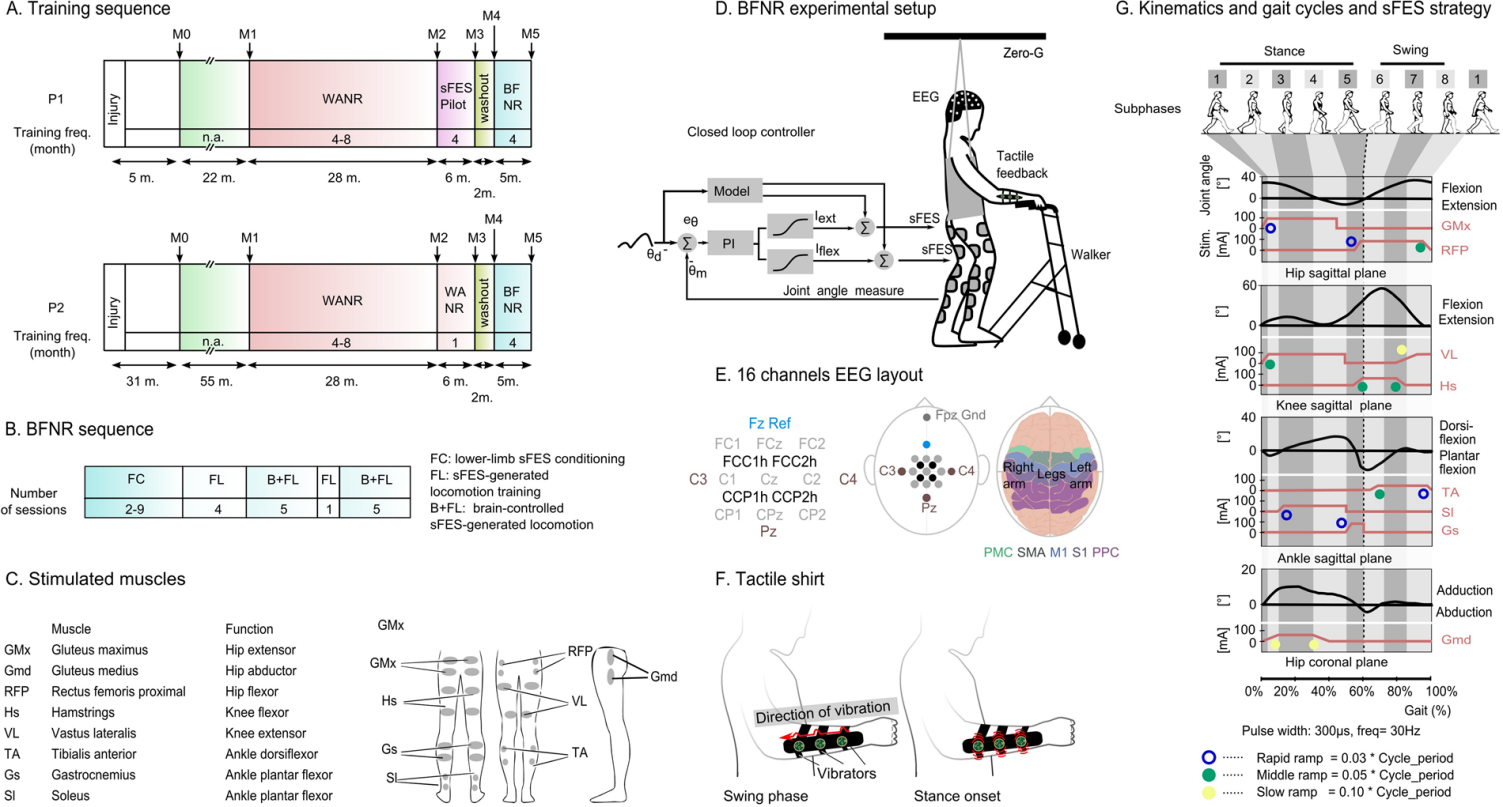
Methodology. (**A**) The timeline for experiment protocol, and six clinical measurements, ranged from M0 to M5. The training frequency (per month) is reported in each cell. WANR: Walk Again Neurorehabilitation Protocol^14,20^; sFES: surface functional electrical stimulation; BFNR: BMI and sFES neurorehabilitation. (**B**) Number of sessions per training phase for the BFNR protocol. (**C**) Eight lower-limb muscles are bilaterally stimulated with the sFES: three muscles for hip mobilization (flexion, extension, and abduction), two for the knee (flexion and extension) and three for the ankle (one dorsiflexor and two plantar flexors). (**D**) Patients were secured by a body-weight support system (Zero-G, Aretech LLC., Ashburn, VA) and used a walker for stability. A stimulation model, containing a reference kinematic profile and gait phases (detailed in subfigure G), was used as a base for the feedforward current stimulation. Patients’ hip and knee angles were measured in real-time. The command of the Proportional Integral (PI) controller was computed based on the error (e_θ_) between the current joint angle (θ_m_) and the reference kinematics (θ_d_). We used a sigmoid function to convert the PI command into feedback currents for the flexors (I_flex_) and extensors muscles (I_ext_). The feedback and feedforward currents were summed to produce the actual values applied to the electrodes. **(E)** EEG electrodes were placed around the medial longitudinal fissure. Channel Fz was used as the reference and the Fpz as Gnd. Arm and leg sensorimotor areas are schematized on the figure on the right; Primary Motor (M1) and Sensory (S1) cortices, Pre-motor Cortex (PMC), Supplementary Motor Area (SMA) and posterior parietal cortex are shown. (**F**) The tactile shirt: six vibrators aligned on the subject’s ulna bone. The subject felt a continuous tactile stimulation going from the wrist to the elbow by the swing phase of the ipsilateral leg and a simultaneous stimulation on all three vibrators at the onset of the stance. (**G**) sFES stimulation pattern for the lower-limb muscles to reproduce the eight sub-phases of a normal gait. The expected range of motion and the proposed stimulation current are shown for hip F/E, knee F/E, ankle dorsiflexion/plantar flexion and hip adduction/abduction. The pulse width and frequency were fixed. Three types of ramps were used to reproduce the gait cycle realistically.

Two individuals with chronic paraplegia due to SCI, designated as P1 and P2 (40 and 32 years old; time since lesion: 4.5 and 10 years) with thoracic level injuries (patient P1 lesion at T7, and P2 at T4) were enrolled in our protocol (Supplementary Table S3). Before the sFES training, these patients had followed the WANR training for 28 and 34 months^14,20^ respectively (Fig. 1A). Both patients had experienced sensory and motor improvements during the WANR and had been converted from AIS A to AIS C by the time of onset of the current protocol. First, we validated our setup for 6 months, for a total of 25 sessions, with patient P1. During this period, we tested our conditioning protocol (12 sessions), validated the closed-loop sFES stimulation (one session), validated the sFES-generated locomotion (10 sessions), and performed the integration of the tactile feedback with our training protocol (two sessions). Next, we tested the BFNR (BMI, sFES, NeuroRehabilitation) protocol with both patients. To isolate the effect of our training protocol, a 2-month washout period was given before the onset of the BFNR (Fig. 1A). The subjects did not receive any locomotion training, nor training in an upright position during this period. Clinical assessments, including the lower-limb circumference perimeter measurement^38^, evaluation of walking function with assistive devices^37^ and neurological status exam^36^ were performed before and after the training protocol for both patients (measurements M4 to M5). We also compared both patients’ neurological improvement rate during the BFNR protocol with their improvement during the WANR protocol (measurements M1 to M2 for P1, M1 to M3 for P2), as well as their spontaneous improvement measured before they started the WANR protocol (measurements M0 to M1). For patient P1, we also controlled for whether our pilot validation tests induced any changes (measurements M2 to M3).

The BFNR protocol was comprised of three basic phases: (1) lower-limb sFES conditioning (FC); (2) sFES-generated locomotion training (FL); and (3) brain-controlled sFES-generated locomotion (B + FL) (Fig. 1B). Patient P2 performed nine FC sessions, followed by four FL sessions, and then 10 B + FL sessions. One extra FL session was recorded after the fifth B + FL session for analyses of patients’ walking aptitude using the sFES. Patient P1, who had already followed a conditioning phase during the pilot validation phase, had two FC sessions followed by the same sequence of FL and B + FL sessions as patient P2.

During all BFNR phases we used a custom 16-channel sFES system^39^ targeting the following muscles in both legs (Fig. 1C): gluteus maximus (GMx), gluteus medius (Gmd), rectus femoris proximal (RFP), hamstrings (Hs), vastus lateralis (VL), tibialis anterior (TA), gastrocnemius (Gs) and soleus (Sl).

During the FL and B + FL phases, patients were partially suspended (65–70%) with a body weight support system (Fig. 1D, Supplementary Fig. S1) (Zero-G, Aretech LLC., Ashburn, VA). During their walking over a four-meter-long linear track, patients assisted themselves with a low-cost walker. The session was supervised by a physiotherapist or a physician. The patients’ hip and knee joint angles were measured every 7 ms. A PI controller adapted the stimulation amplitude in real-time to the muscle conditions determined by the lower-limb joint angles measurements. Two additional elements completed the setup: (a) a 16-channel EEG electrode (Fig. 1E) placed around the medial longitudinal fissure, densely clustered over the putative leg sensorimotor cortical area to detect the cortical activation for leg motor imagery; and (b) a portable haptic device^26^ that permitted the patients to perceive the position of the stimulated leg on their upper-arm (Fig. 1F). Continuous waves of tactile vibrations delivered to the patients’ forearms coincided with the stimulation of the lower-limb muscles to indicate the beginning and end of the swing phase of a stride.

The preparation, followed by the training session, required approximatively 95 minutes, divided as follows: the sessions started with 15 minutes stretching, targeting both the upper-limbs, trunk, and lower-limb muscles. Next, approximately 35 minutes were used during the session preparation (placement of the EEG and sFES electrodes, placement of the position sensors and portable tactile feedback, placement of body weight support harness and moving patient to an upright position). The training lasted 45 minutes. We developed a novel sFES stimulation strategy that used time-based muscle contractions considering the eight sub-phases^40^ of a physiological gait pattern^41,42^ (Fig. 1G): (1) initial foot contact; (2) load response; (3) mid-stance; (4) terminal stance; (5) pre-swing; (6) initial swing; (7) mid-swing; and (8) terminal swing. To reproduce adequate gait during each sub-phase, we fine-tuned the stimulation time, the stimulation ramps up/down, the amplitude and the frequency for each muscle.

### PI controller validation

**Figure 2:**
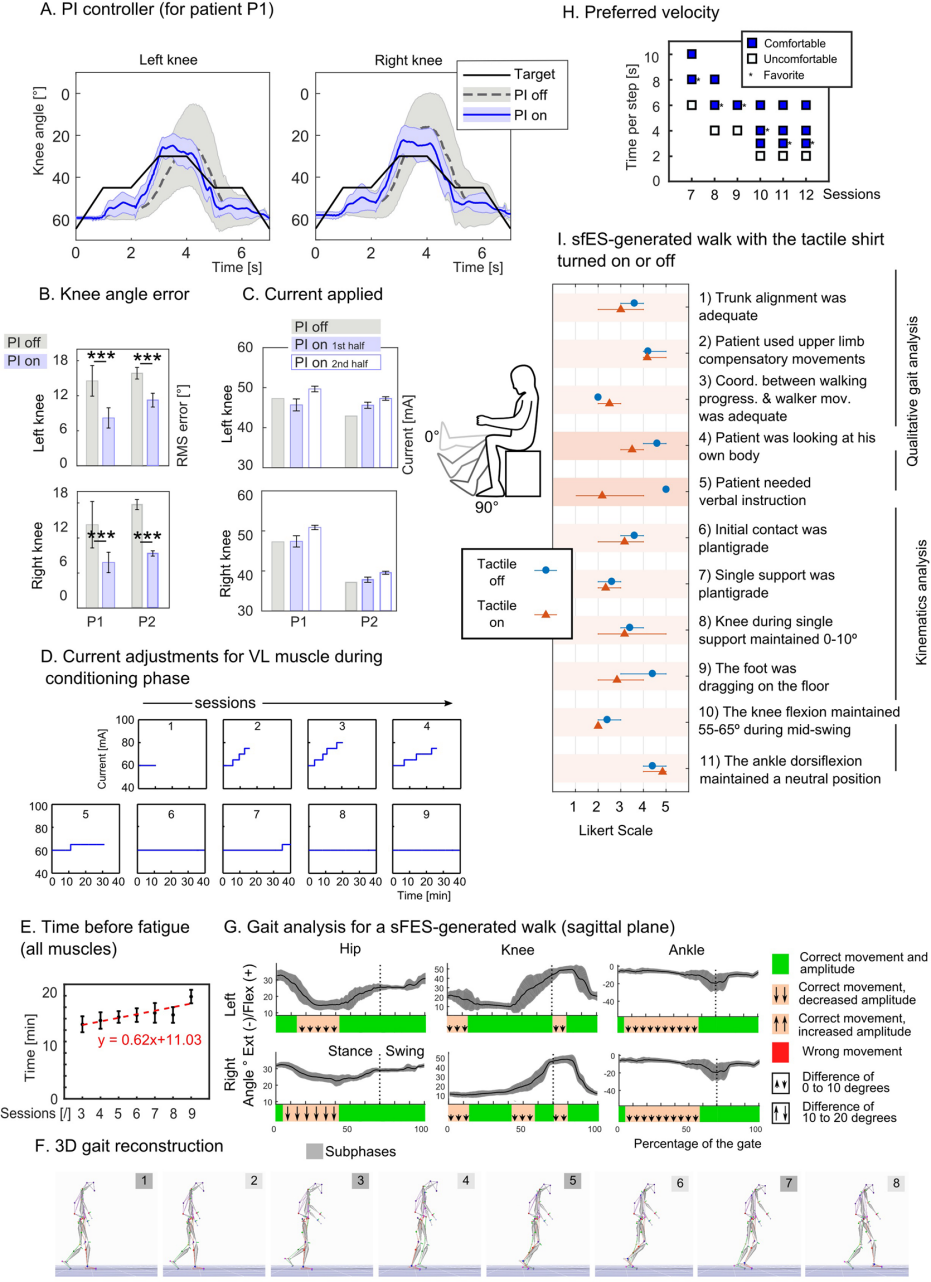
Setup validation. (**A**) Validation test of the PI controller; range of motion of the knee joint with and without the PI controller during a knee extension task with P1. We compare the joint angle produced by the controller in both cases (mean ± SD, n = 56) to the expected target. (**B**) Root mean square error between the desired and measured knee angle (mean ± SD) for both patients. Two-sided two-sampled t-test is reported. (**C**) Current applied without PI controller and with PI controller for the first and second half of the attempts (mean ± SD, n = 56). (**D**) The amplitude of current for the vastus lateralis (VL) muscle during the conditioning sessions for patient P1. The duration of the session was set to 10 minutes and increased by 5 minutes every session (max. 40 min). When the therapist observed a clear sign of fatigue, the current was increased by five mA. The initial amplitude of a session was always set to 60 mA for this patient. (**E**) Mean ± SEM of the time between two changes of current amplitude, considering all muscles, during the first 20 minutes of conditioning sessions three to nine for patient P1. Red line shows the linear regression of the means and corresponding linear function. (**F**) 3D reconstruction of the gait based on motion capture data measured during an FL session with P2, and (**G**) angles for hip, knee ankle in the sagittal plane (mean + − SD over 54 full gait cycles). The lower part of the graph reports if the movement was correct (green) or not (orange or red). We report in orange the period where the movement was correct but the amplitude of the movement smaller (arrows pointing down) or higher (arrows up) than expected (small or big arrows describe if the difference was respectively in the 0–10° or 10–20° range). (**H**) Self-reported sets of comfortable walking speeds for patient P1. Walking speed varied on each run and the patient was asked to report if the speed was comfortable or not and if it was his favorite speed of the current session. (**I**) An experimented physiotherapist, blinded to the experimental conditions used an 11-item visual evaluation scale on qualitative and kinematics’ aspects of patients’ P1 and P2 sFES-generated walk when the tactile shirt was turned off (blue dot) or turned on (orange triangle).

We introduced a PI controller to adapt the electrical stimulation pattern of the sFES system, based on muscle responses, to generate a reliable gait and cope with variance regarding muscle responsiveness and fatigue. To illustrate this effect, Fig. 2A compares the knee angle for patient P1 during a sFES-generated knee extension task in a seated position, when the PI controller was turned on (blue line) or off (grey line). To guarantee well-controlled conditions, only the knee joints were controlled for this test, while the other joints remained immobilized. In both conditions, the same desired target angle (black line) is used for comparison. The test was repeated with both patients. We found that the root mean square error (RMS) for the PI-on condition (left knee 8.2°, right knee 7.8°, for P1, 11.2° and 9.3° for P2) was significantly smaller compared to PI-off (left knee 14.5°, right knee 14.4° for P1, 15.5° and 17.6° for P2) (two-sided two-sample t-test attests the significant difference (t(110) = 15.13, p = 0 for P1; t(110) = 15.20, p = 0 for P2) (Fig. 2B). These findings confirm that the PI controller produced more reproducible responses to the patients even in the presence of muscular fatigue.

To cope with the increase in fatigue, the PI controller increased the amplitude of the stimulation for the last part of the sessions (Fig. 2C). The mean current employed was only 6% higher than in the PI-off condition for P1 and 7.8% for P2. Considering the entire session, the mean currents for both conditions were comparable for both P1 (mean current of 49 mA for PI-on and 47 mA for PI-off), and P2 (42.51 mA PI-on, 39.96 PI-off), suggesting that the use of the PI did not notably increase the necessary current for stimulation.

### sFES-Conditioning validation

The FC phase was introduced to delay the onset of muscle fatigue while increasing muscle responses. Three factors were gradually adjusted during this phase: the stimulation time, the amplitude and the number of muscles stimulated simultaneously. The first training session was set to 10 minutes whereas the following sessions were gradually increased up to 40 minutes. At the onset of the sessions, the stimulation amplitude was set to the minimum current that produced a clear muscle contraction. Then, if the therapist observed a notable decrease in a given muscle response, the corresponding stimulation current was increased by 5 mA (maximum: 100 mA).

An example of stimulation amplitude is shown for nine sessions of VL muscle for patient P1 (Fig. 2D). For session three, during the first 20 minutes of training, it was necessary to increase the current five times (from 60 mA to 80 mA) to observe a muscle activation that led to an overt movement. On the following day, at the same moment throughout the session, only 70 mA were needed to generate the same response. This positive trend continued throughout the training. Thus, after the sixth session of conditioning, the patients’ VL muscle response did not decay after 40 minutes of stimulation at 60 mA.

We calculated the mean time before fatigue for all muscles during the first 20 minutes of the session. Considering only sessions that lasted 20 minutes or more (to avoid bias due to the session length), we observed a significant linear increase of the average time before fatigue (Fig. 2E, y = 0.62x + 11.03, P = 0.01), confirming that the patients’ muscle responses improved following FC sessions.

### sFES stimulation strategy validation

Gait-analysis based on 3D construction of motion capture data of patient P2 revealed that the majority of the markers of the physiological walk were present (Fig. 2F, detailed analysis in Supplementary Fig. S2, Supplementary Video S1). Indeed, on initial contact, we observed the accurate hip flexion, and the expected neutral position for the knee and the ankle. Through the mid-stance phase, we observed an accurate, neutral position for both the hip, knee and ankle joints. For the pre-swing, as expected, we observed the flexion of both the hip and the knee and the ankle plantar-flexion. We documented the flexion of the hip and the knee during the initial swing, and a neutral position of the ankle during the mid-swing. On the terminal swing, the hip flexion was correctly maintained, the ankle was in neutral position, and the knee extended.

In three cases the range of motion was smaller than the normal gait: hip extension during the terminal-stance, the second peak of knee flexion during the swing phase, and the ankle dorsiflexion during mid and terminal stance. The hip extension and the ankle dorsiflexion limitations reduced the overall step length, whereas the limitation on the knee flexion reduced the foot clearance during the swing. Nevertheless, none of these limitations was severe enough to compromise the patient’s gait.

We also wanted to be sure to use the fastest walking pace, to maximize the number of steps per session, while guaranteeing a comfortable and safe training environment for the patients. We performed a systematic evaluation of the maximum comfortable speed during six sessions with patient P1. For each run of 12 steps, we varied the speed and asked the patient whether the run was comfortable or not and whether the run was the patient’s favorite among the ones achieved in the current session. The patient was told before the session that the speed could change from one run to another, but no further information was given during the session. Over time, the patient reported feeling comfortable with speeds that were gradually increased (Fig. 2H); while the threshold for comfortable speed was between 6 and 8 seconds per step (s/step) during session seven, it reached a maximum of 3 s/step on the 10^th^ session. By then, the preferred speed improved from 8 s/step to 3 s/step.

### Haptic feedback validation

SCI induces a loss of tactile and proprioceptive feedback information that is needed to generate a correct walking pattern^43,44^. To overcome this limitation, we integrated a portable haptic device, developed by our group (named the tactile shirt)^26^, composed of arrays of vibrators to display relevant events using a tactile illusion called the apparent movement^45^.

We ran two sessions with patient P1 to evaluate the difference of behavior in the presence or absence of the tactile feedback (Fig. 2I). An expert physiotherapist, blinded to the conditions of the experiment used an 11-item visual assessment developed by our team to evaluate differences in terms of the patient’s adaptation to our locomotion training setup (questions 1–5) and the patient’s gait kinematics during the sFES-generated locomotion (questions 6–11) (Supplementary Table S4 for details). The presence of the tactile shirt did not positively or negatively influence the patient’s trunk alignment, his need for compensatory movements, or his coordination with the walker. As expected, neither did we observe any substantial difference in any of the kinematic-related questions: the presence of the tactile feedback did not change the patient’s muscle responses during the stance (questions 6–8), or the swing (questions 9–11) phases.

On the other hand, we observed that the patient developed a tendency to look less at his own body during the walk when the tactile feedback was present (question 4). This phenomenon allowed patients to express a better understanding of the position of the leg in space, as well as the movement of the lower limbs. During the sessions, the therapist instructed the patient about correct trunk posture, upper and lower-limb participation, as well as the best way to coordinate the walker. We noticed a reduction in the need for these verbal instructions when tactile feedback was employed (question 5). Overall, we found that tactile feedback was useful for the patient to perform the task more independently.

### BFNR validation

After completing the pilot validation tests, we applied our BFNR protocol to both patients. Here, we present a quantitative and qualitative analysis of the patients’ walking with our sFES system, their performance with the BMI and their performances with the setup integrating the BMI and the sFES. Overall, both patients managed to walk correctly using the sFES and the stimulation paradigm proposed in this project (see Supplementary Movies S2 and S3 for runs with both patients).

**Figure 3:**
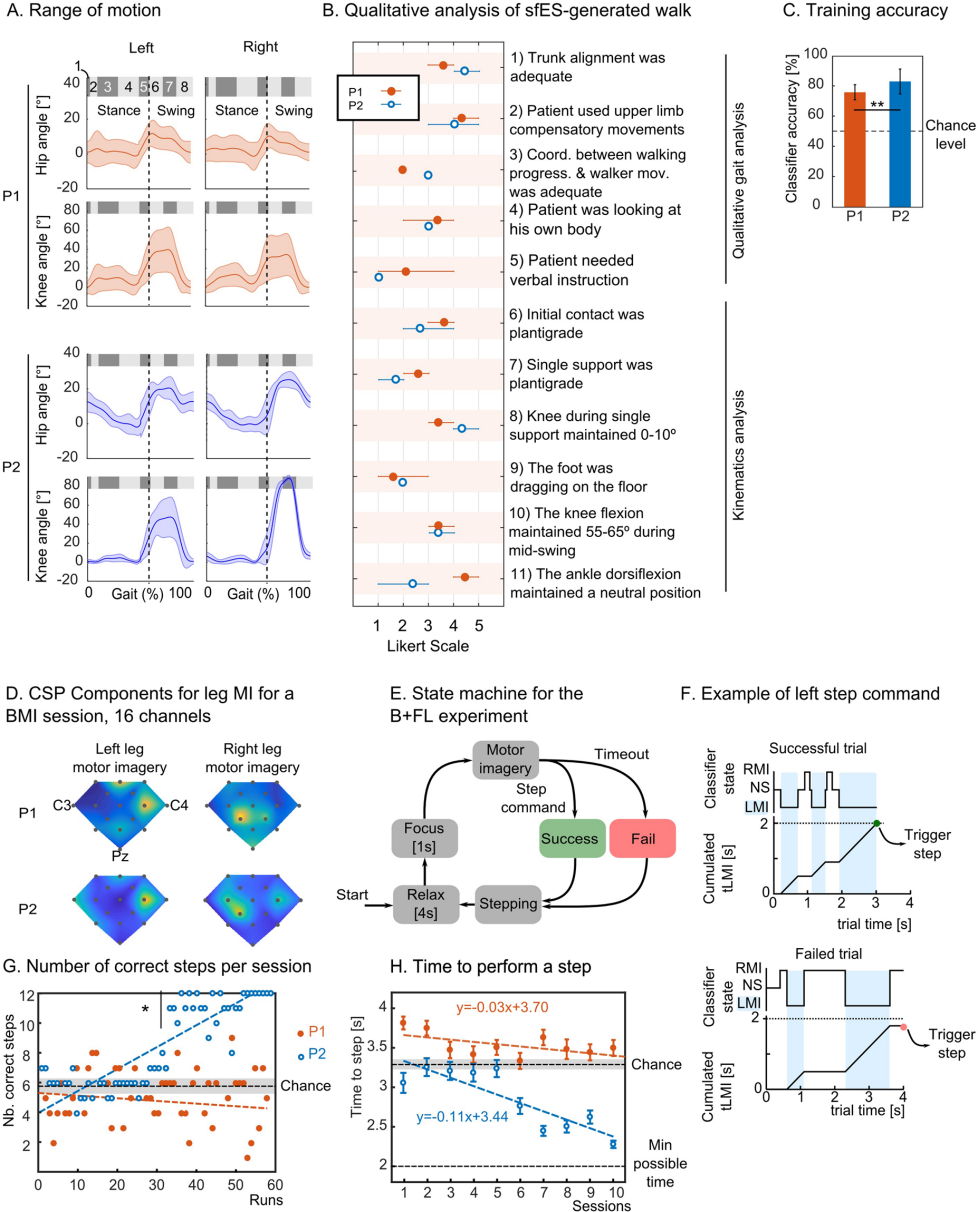
Protocol validation. (**A**) The ranges of motion for hip and knee joints for both patients. The eight gait subphases are shown inside the gray boxes. Dashed line shows the onset of the swing phase. (**B**) Qualitative and kinematics’ aspects of patients’ sFES-generated walk. **(C)** Patients’ classifier accuracy for the EEG training phase (mean ± SD, on the 10 B + FL sessions and ttest). **(D)** The coefficient of the principal component found by the CSP algorithm for a B + FL training session for left and right leg motor imagery. Electrode placement is the same as in Fig. 1E (three electrode names C3, C4 and Pz are shown as references). **(E)** For each step, the following sequence was reproduced: the patient relaxed and adjusted himself for 4 seconds, focused during 1 second and had 4 seconds to produce the motor command. During this window, if the patient maintained the correct motor imagery for 2 seconds, the step was triggered and considered as successful. Otherwise, if the timeout was reached, an automatic step was triggered, and the trial was considered as failed. **(F)** Example of successful and failed step. RMI/LMI: right/left motor imagery, NS: no state, t_LMI_: cumulated time of left motor imagery **(G)** Number of correctly performed steps for all patients for all B + FL runs over 12 steps. The chance level is reported with black dashed lines, 95% interval of confidence is shown in gray. **(H)** Mean ± SE time to control a step with the B + FL protocol. Each dot represents 12 steps of a run. Fastest possible time to perform the task was 2 seconds, and the chance level is shown with a black dashed line, 95% interval of confidence in gray (3.23–3.37 [s]). Linear regression is represented by dashed lines for each patient.

To have a complete view of the behavior of the two patients with our setup, we carried out a qualitative analysis of the patients’ sFES-generated walking patterns during their last FL sessions. Figure 3A shows the hip and knee range of motion for these sessions and Figure 3B the detailed study carried out by a trained physiotherapist blinded to the experiment.

Considering the metrics for high-level coordination, we did not find notable differences between the patients; both patients had good trunk alignment during the tasks; they, nevertheless, used compensatory movements to maintain their upper body. As patients used the tactile shirt in both sessions, we found results consistent with the pilot tests for P1. Basically, both patients did not need verbal instructions, nor did they need to constantly look at their bodies during the sFES-generated locomotion. During the stance phase, both patients had good knee position but poor plantigrade position during single support. Patient P1 had expected responses during the swing phase, with his foot rarely dragging on the floor. Patient P2 had difficulties in this aspect of the swing phase, due to a smaller response of the tibialis anterior muscle. We hypothesize that this lack of muscle response was due to an observed tension in this patient’s gastrocnemius (Gs) muscle (which is the antagonistic muscle of this movement). Likely, this resulted from his positioning in his wheelchair since his knees and feet usually stayed flexed in his wheelchair, which could lead to increased tension in the Gs muscle.

Both patients in this protocol had been previously trained with the BMI protocol using an arm or leg motor imagery as described in the WANR protocol^14,20^ for over 2 years. However, the specific BMI protocol proposed in the current study was new to both patients. Each B + FL session started with an 8-minute open-loop training block where patients were randomly instructed to imagine moving their left or right leg. The recorded EEG data was used to train the EEG classifier. The classification accuracy for both patients was above chance (73.9% ± 3.6% for P1 and 83.2% ± 8.2% for P2) (Fig. 3C). Patient P2’s classifier accuracy was significantly higher than for P1 (t-test, P = 0.002). To confirm that patients effectively used leg motor imagery during the B + FL sessions, we analyzed the principal components calculated with the common spatial pattern filter (CSP)^46^ (Fig. 3D). The CSP algorithm detects the EEG electrodes that maximize the variance for the two classes (here, left leg versus right leg motor imagery). For left leg motor imagery, we found the main activation over the C2 electrode for both patients (see Fig. 1E for electrode placement), while for right leg motor imagery, the CSP filter detected the highest variance over electrodes CCP1h, confirming cortical activation in the leg motor area.

Next, patients learned to perform leg motor imagery to trigger the stimulation of the corresponding leg through an EEG-based brain-machine interface (Fig. 3E). An array of LEDs informed the patients of the current state of the trial and the brain activity classification. A trial was defined as follows: after a relax period of 4 seconds where the patients could adjust themselves, and 1-second focus time, the patients had a 4-second time window to perform the motor imagery (Fig. 3F). If the patient managed to maintain the correct motor imagery for 2 seconds, the trial was considered as successful, and the corresponding step was triggered. If the timeout was reached, the trial was registered as a failure, and an automatic step started. The time to trigger a step, therefore, ranged between 2 and 4 seconds. During each session, patients performed 72 steps divided into six runs separated by a 1-minute break.

The number of correct steps per run is reported in Fig. 3G. Patient P2 outperformed patient P1 regarding the number of successful trials per session by performing 88% of the runs above chance and more than one-fifth of the runs with a perfect score (Fig. 3G). Throughout the training, we observed a clear improvement in terms of control accuracy for patient P2. Indeed, when considering the second half of the training, 25 out of 30 runs were significantly better than chance (mean + 2*SD).

Patient P1 had just 50% of the runs above chance level. This shows that the BMI protocol and the need to maintain the leg motor imagery for 2 seconds was particularly difficult for patient P1 who, despite having a good classifier score (on average >70%), did not manage to perform the BMI control above chance level.

We also analyzed the mean time to perform a step for each session (Fig. 3H). We found a small but statistically significant trend towards reducing the time to perform the task in patient P2 (*y* = −0.11*x* + 3.44, *p* = 0) and patient P1 (*y* = −0.03*x* + 3.7, *p* = 0.03) with training. Altogether, we observed significant control performances in patient P2. Patient P1, who started with lower performance rates, improved (although at a slower rate) with the training and could have potentially reached better control with more extended BMI training.

### Clinical improvements

Following the BFNR protocol, we observed notable physical improvements in both patients. This was first verified by an increase in muscle volume through the perimeter assessment^38^. We found, as expected, an increase of the thigh volume of 276 cm^3^ in patient P1 and the lower-leg volume of 636 cm^3^ (Table 1, Supplementary Fig. S3). Patient P2 also exhibited this effect, with an increase of 838 cm^3^ of thigh volume and 280 cm^3^ for the lower-leg volume.

**Table 1:** Difference between the post-BFNR (M5) and Pre BFRN (M4) measurements for the lower-limb perimeter.

	P1	P2		
	Perimeter diff (cm)	Volume diff (cm^3^)	Perimeter diff (cm)	Volume diff (cm^3^)
Hip	−0.7	276	2.1	838
	1.0		1.4	
	1.1		2.6	
	0.6		3.5	
	0.1		3.7	
	0.2		2.05	
Knee	2.5		0	
	0.8	636	1.6	280
	0.4		1.8	
	2.5		2	
	3.9		1.7	
	4.3		1.15	
	2.5		0	
Ankle	−3.1		−2.75	

We next analyzed the patients’ walking functions using assistive devices. For this, we employed the WISCI II evaluation^37^, which assesses the amount of physical assistance needed, as well as the required devices (orthosis and gait assistive devices), for a patient with SCI to walk 10 meters (Table 2). The exam was performed with no body weight support and no sFES. At the onset of the BFNR (M5), patient P1 was able to walk 10 meters using a lower-limb orthosis (HKAFO: hip-knee-ankle-foot orthosis), a triangular walker and required additional physical assistance for hip and trunk stabilization (WISCI score of 6). Following the BFNR protocol (M6) this patient’s functional score improved to 9, as he required no physical assistance to perform the task. He also managed to perform the same test with crutches and one assistant (score of 7). Additionally, P1 was able to accomplish the task 19 seconds faster than at the onset of the BFNR. No notable changes were observed during the pilot validation tests (M3 to M4). Considering the necessary level of assistance to perform the 10 m task, we did not register changes for patient P2 (score of 6 at the onset and the end of the protocol), he nevertheless managed to perform the test 14 seconds faster.

**Table 2:** Walking functions assessment with assistive devices with the Walking Index for SCI (WISCI II) evaluation^37^.

	P1				P2	
	M3	M4	M5	M6	M5	M6
**WISCI**						
Score	6	6	6	9	6	6
Time to walk 10 m [s]	99	98	108	89	66	52
**Vital signs (pre)**						
PA (mmHg)	130 × 90	120 × 80	130 × 90	120 × 80	120 × 70	120 × 80
Heart rate (bpm)	94	92	81	74	79	76
Sat O_2_ (%)	99%	97%	96%	97%	96%	96%
Borg^a^ (Upper limbs)^b^	0	0	0	0	0	0
Borg (Respiratory)^c^	0	0	0	0	0	0
**Vital signs (post)**						
PA (mmHg)	130 × 85	120 × 80	130 × 90	130 × 80	130 × 80	130 × 70
HR (bpm)	124	93	96	110	120	99
Sat O_2_ (%)	98%	92%	97%	99%	99%	98%
Borg (Upper limbs)	3	4	5	3	2	2
Borg (Respiratory)	2	2	5	3	2	1

We evaluate the time to perform the 10 m task and vital signs recorded before and after the test. ^a^The Borg fatigue assessment is a subjective self-reported level of exertion during exercise; ^b^Level of arms fatigue; ^c^Measurement the general physical fatigue (heat, sweat or dyspnea).

Both patients experienced a small decrease in the resting heart rate (HR) throughout the protocol (81 bpm to 74 for P1, 79 bpm to 76 for P2, Table 2). Similarly, the HR increase due to the effort was smaller at the end of training for P2 (HR increase of 41 bpm at the onset of the training (M5 and 23 bpm at the M6). Patient P1 improved during the pilot tests (an increase of HR of 30 bpm was observed at M2, and an increase of 1 at M3). During the measurement following the BFNR, this patient managed to perform the task with less assistance which translated into a higher increase in HR (36 bpm).

The Borg fatigue assessment is a subjective self-reported level of exertion during exercise ranging between 0 (no fatigue) to 10. Patients were asked to report the level of fatigue in their upper-limbs. We observed a small decrease in the fatigue assessment following the BFNR for patient P1. Blood pressure and oxygen saturation were also measured before and after each WISCI assessment (Table 2). All recorded values were in the normal range.

**Figure 4:**
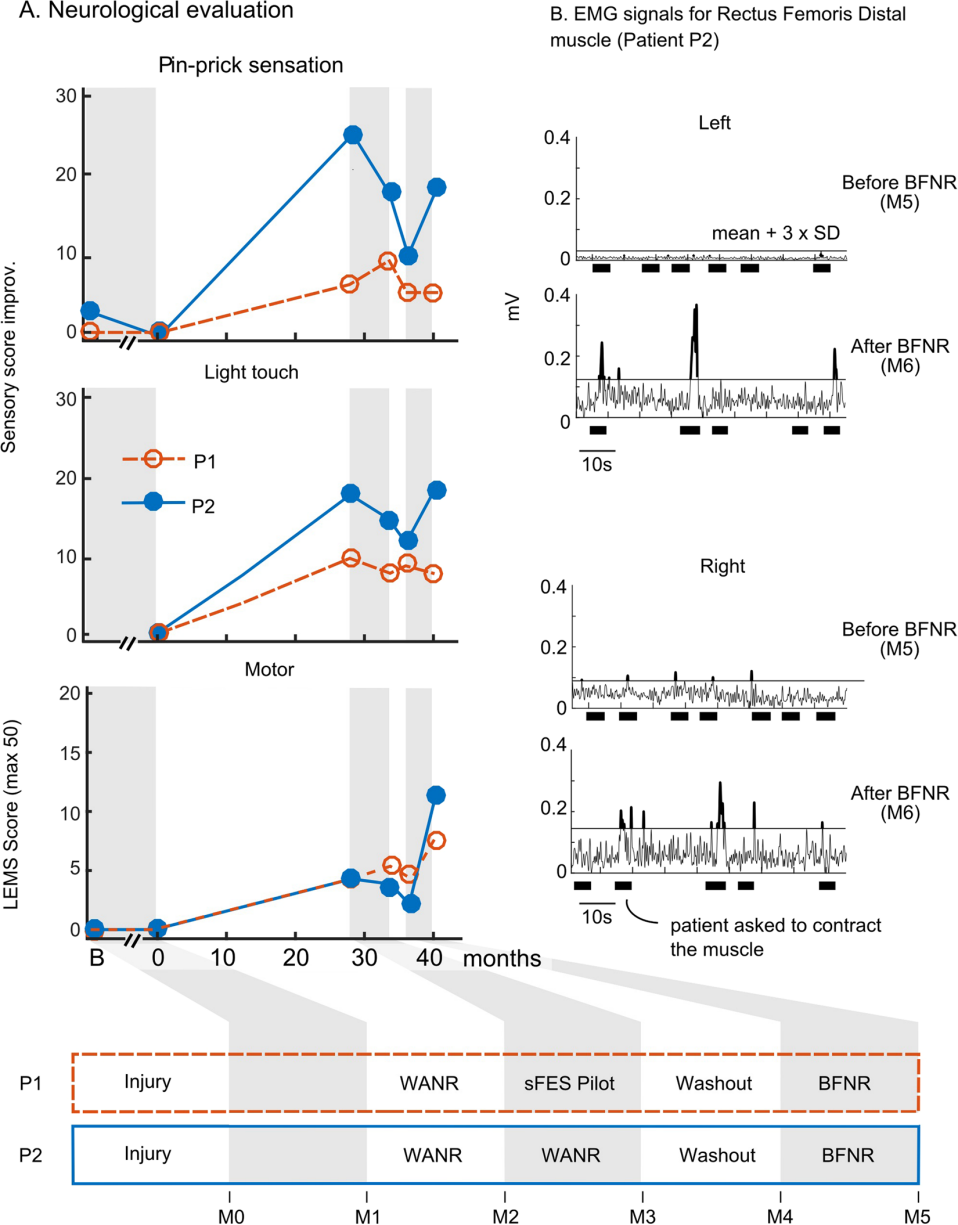
Neurological improvements. (**A**) Six neurological evaluations (M0 to M5) for both patients. Sensory score improvement is calculated using standard ISNCSCI assessment, compared to the score at the onset of the training (for both patients, the score at time = 0 was subtracted from all evaluations). The lower extremity motor score (LEMS) is based on clinical evaluation of five key lower-limb muscles (0 for no contraction, 5 for normal), the maximum score is 50. (**B**) Rectus muscle EMGs for patient P2 before and after the BFNR protocol. Signal was filtered between 5 Hz and 250 Hz with a Butterworth 4^th^-order band-pass, and a 60 Hz notch filter. The envelope was obtained after rectification of the filtered signal and a 1 Hz Butterworth high-pass filter. Thick black lines represent the physiotherapist’s instruction time to contract the muscles. The black horizontal line is the mean + 3 x SD of the activation as calculated during the baseline periods.

No clear neurological improvement was observed in the patients’ sensory domain (light touch, pin-prick sensation) during the BFNR (Fig. 4A). Patient P2 had an improvement rate comparable to the one he had during the first 28 months of WANR, while patient P1 stagnated.

On the other hand, we documented significant motor improvement in both patients. The improvement was assessed through the lower extremity motor score measurement (LEMS) of five lower-limb muscle groups (rectus femoris proximal and distal, tibialis anterior, extensor hallucis longus and gastrocnemius). For a complete view, we recorded seven additional lower-limb muscles (hip adductors, gluteus maximus, gluteus medius, medial and lateral hamstring,flexor hallucis longus and extensor digitorum longus). The scoring method followed the standard ASIA motor exam conventions, ranging from 0 (no contraction) to 5 (normal contraction). We show the LEMS score for both patients in the lower panel of Fig. 4A, and the details per muscle in Table 3. For patient P1, we observed, a 1 point LEMS increase during the sFES pilot phase and a gain of 3 points during the BFNR phase. This was an encouraging result, as it demonstrated that even at the chronic phase of an SCI, it is still possible to observe neurological improvement. Notably, patient P2 exhibited a much higher motor improvement (Supplementary Movie S4); starting with an LEMS of 2 measured at the onset of the BFNR training. This patient reached a final score of 11 points. Considering all 12 muscles, this patient went from a score of 6 to 21 (Table 3). By the end of the training, patient P2 could contract the Rectus Femoris Distal (RFD) muscles with a score of 2. This was confirmed both clinically and thorough EMG recordings (Fig. 4B) showing the presence of significant contractions at the end of the BFRN.

**Table 3:** Muscle score for five key muscles^36^ and seven auxiliary muscles.

	Muscles			P1								P2			
				M3		M4		M5		M6		M5		M6	
	Function	Main muscles		Pilot tests				BFNR				BFNR			
				R	L	R	L	R	L	R	L	R	L	R	L
**Key muscles**	Hip flexor	Iliopsoas, rectus femoris proximal		1	1	1	1	1	1	1	1	0	0	1	1
	Knee extensor	Vastus, rectus femoris distal		1	1	1	1	1	1	1	2	1	1	2	2
	Ankle dorsiflexor	Tibialis anterior		0	0	0	0	0	0	0	0	0	0	1	1
	Hallux extensor	Extensor hallucis longus		0	0	0	0	0	0	0	0	0	0	0	1
	Ankle plantar flexor	Gastrocnemius, soleus		0	0	1	0	0	0	1	1	0	0	1	1
**Auxiliary muscles**	Hip adductor	Adductors		2	2	2	2	1	2	1	1	1	1	1	1
	Knee flexor	Medial hamstring		0	0	0	0	0	0	0	0	0	0	1	1
	Knee flexor	Lateral hamstring		0	0	0	1	0	1	0	1	0	0	1	1
	Hip abductor	Gluteus medius		1	1	1	1	1	1	1	1	0	0	1	1
	Hip extensor	Gluteus maximus		2	2	1	1	1	1	2	2	1	1	1	1
	Toes extensor/flexor	Extensor/flexor digitorum longus		0	0	0	0	0	0	0	0	0	0	0	0
	Hallux flexor	Flexor hallucis longus		0	0	0	0	0	0	0	0	0	0	0	0
		**LEMS (sum key muscles)**		**4**		**5**		**4**		**7**		**2**		**11**	
		**Sum all muscles**		**14**		**14**		**12**		**16**		**6**		**21**	
		**sFES muscles (16 muscles)**	**Regression**	2				0				0			
			**Stagnated**	14/16				11/16				2/16			
			**Improved**	2/16				5/16				14/16			
		**Non-sFES muscles (8 muscles)**	**Regression**	0				1/8				0			
			**Stagnated**	8/8				7/8				7/8			
			**Improved**	0				0				1/8			

The test is done without any sFES stimulation. The muscles that were targeted during the BFNR training are in underlined. The lower part of the table shows the number of muscles among the ones that were targeted (sFES muscles) and the ones that were not targeted with the sFES (Non-sFES muscles) that have regressed, stagnated or improved during the pilot test and the BFRN.

We next analyzed the proportion of muscles that improved with the training, considering the muscles that were stimulated with the sFES separately from those that were not (lower part of Table 3). For patient P1, no clear improvement was observed during the pilot tests. During the BFRN for this patient, none of the non-stimulated muscles improved, but 5 out of the 16 stimulated muscles improved. For patient P2, we observed an improvement in one out of the eight non-stimulated muscles and 14 out the 16 stimulated ones. We observed that the best motor improvement occurred in muscles that were stimulated with sFES, with notably better responses for patient P2.

Overall, the motor improvement in both our patients could not be compared to the type of spontaneous improvement rates reported in the literature (scores <1 point when the training starts 26 weeks after the injury^2^) or those recorded for our patients prior to the start of training in our laboratory (0 for both patients, M1-M0). For patient P2, the improvement observed following the BFNR (9 points after just 5 months) was even higher than the one he experienced during the WANR protocol (i.e., a 4 point improvement during the first 28 months).

## Discussion

This paper introduces a novel, non-invasive neurorehabilitation protocol for locomotion training for patients with severe chronic paraplegia resulting from spinal cord injuries, which targets both musculoskeletal training and corticospinal plasticity. Our setup integrated several technical novelties: a new sFES paradigm targeting muscle activation to produce locomotion, a BMI paradigm based on detection of left and right leg motor imagery to trigger the sFES, and sensory substitution to provide patients with tactile feedback from the lower limbs. We also introduced a step-by-step conditioning/training protocol and validated it with two individuals with paraplegia.

Using this novel approach, we successfully tested a stimulation protocol to reproduce a smooth gait pattern; patients were able to use our setup to walk with partial suspension and little or no external help. This result was possible thanks to muscle conditioning preceding the sFES-generated walk. Following an SCI, muscles with myotomes originating below the lesion level undergo severe disuse atrophy^47,48^. Several years after the lesion, the volume of muscle fibers is reduced, becoming more and more prone to the process of fat infiltration. Concurrently, slow fatigable fibers tend to transform into fast fatigable fibers^47^. As a result of our training protocol, our patients experienced an increase in the volume of their leg muscle fibers and a parallel increase in their resistance to muscle fatigue^21,49–51^.

Another important aspect of our strategy was the stimulation of a large number of muscles (16 lower-limb muscles) with precise timing. This was possible thanks to a custom-built programmable sFES stimulator^39^ to target each muscle individually. The conventional sFES technique to reproduce locomotion uses stimulation of the peroneal nerve to elicit the triple-flexion reflex during the swing and stimulation of the gluteus and the quadriceps during the stance phase of the gait^35,52,53^. This technique is limited by the high variability of the muscle response and the rapid habituation to the stimulation^24^. Instead, in our protocol, we decided to directly stimulate the lower-limb muscles to produce a consistent gait pattern. A major technical difficulty of our approach came from the inherent non-linearity of muscle responses^54^ (sigmoidal shape response type) and the effect of muscular fatigue due to the recruitment of muscles fibers in a non-selective, spatially fixed and temporally synchronous pattern^55,56^. In SCI subjects, following the lesion, there is also a trend towards the substitution of muscle fibers into rapidly fatigable fibers^47^. To cope with these difficulties, we proposed and successfully tested a closed-loop sFES controller using the joint angles of the lower-limbs to adapt the stimulation in real time. We also introduced online tactile feedback to help patients perceive gait events. Previously, our group has reported that this sort of feedback promotes cortical plasticity^26^ and provides patients with the type of lower-limb sensory information needed to improve their gait. Here, we observed that the use of tactile feedback increased the patients’ confidence and independence during the walk. Overall, using our setup and following our protocol, our patients acquired the ability to produce a smooth sFES-generated gait pattern. Indeed, for both patients, the main clinical features of human gait were respected: absence of crouching at stance, neutral position of the ankle at initial contact and during stance phase, as well as sufficient foot clearance during the swing.

Our BFNR protocol was specifically designed considering the use of BMI for neurorehabilitation purposes (rather than assistance)^35,57^ for patients with SCI with minimal or no motor function. Therefore, the focus was not placed at achieving the highest level of control accuracy or speed, but rather to create a condition in which both afferent and efferent signals, converging at the level of the lesion, are engaged simultaneously. Thus, to trigger a step, patients had to perform and maintain leg motor imagery of the corresponding leg for two seconds. This protocol was particularly complicated for one patient who experienced more difficulties in control performance. Future work with a larger cohort of patients is necessary to address this issue; for example, a shorter trial-time could potentially facilitate the task.

As the final outcome of this study, we observed that patients exposed to our protocol exhibited important clinical improvements, including, as expected, increases in muscle volume^58^. Moreover, we observed better outcomes in walking functions using assistive devices. As a result, our patients’ step frequency increased, and one patient was able to walk using less assistance at the end of the protocol. This was probably a consequence of better overall physical condition, which was indirectly inferred by the decrease in resting heart rate, smaller heart rate increase after the effort, improved muscular resistance, better coordination with walking devices due to the use of a walker and body weight support in the protocol, and the occurrence of a significant partial neurological recovery in the motor domain. Considering this latter component, partial motor neurological recovery was observed in different degrees: it was moderate in patient P1 and substantial in patient P2. Importantly, P2 was the patient who had good BMI control and good sFES responses. Further clinical trials are necessary to disambiguate if achieving good BMI-control accuracy is a necessary condition or even a determinant for patients to benefit from this training.

Yet, there is one major question that remains open: what is the mechanism that induces this improvement? We hypothesize that, like in the case of our previous results with the WANR, the concurrent occurrence of cortical plasticity, induced by BMI use^11,13,14^ and spinal plasticity, promoted by sFES through ascending signals^59–61^ was an essential agent for the observed neurological motor improvement in our study. Recent work with human subjects showed that the use of invasive epidural stimulation has the potential to induce partial neurological restoration in chronic complete SCI patients^9^. Importantly, a study with rats^15^ showed that the motor recovery was faster and more important when the epidural stimulation was paired with brain-machine interfaces compared to conditions where epidural stimulation was used alone. These findings provide clear support for our hypothesis.

For both patients, the cortical activation during leg motor imagery was found close to the cortical leg sensory-motor area. The presence of activation in posterior areas was previously reported in patients with chronic complete SCI due to cortical reorganization towards the primary somatosensory cortex^62–64^.

Given the improvements obtained in this pilot study in terms of muscular volume and performance, better walking functions and neurological recovery, we propose that our BFNR protocol has the potential to become a valuable neurorehabilitation therapy for patients with SCI in the future.

## Methods

### Patients inclusion/exclusion criteria

Subjects were adults diagnosed with paraplegia, with traumatic SCI thoracic injury at the chronic phase of the lesion, emotionally stable and with the absence of offset comorbidities. The inclusion criteria for the present study considered patients that had previously participated in the WANR protocol and who exhibited evidence of upper motor neuron injury, clinically manifested by the presence of spasticity and positive response to spinal reflexes. The screening was performed to test their muscles’ responsivity to sFES before inclusion. Exclusion criteria included lower motor neuron injury, the absence of muscle response during sFES responsivity screening, a degree of spasticity exceeding a score of 2 (Ashworth scale), and a degree of osteoporosis (T-score) greater than −4. Two patients were included in this study. Prior to the intervention, patient P1 and P2 followed a neurorehabilitation protocol introduced by our team (Walk Again Neurorehabilitation Protocol, WANR)^20^ which integrated the use of body weight support (BWS, Zero-G, Aretech LLC., Ashburn, VA), Lokomat^6^, brain-machine interface (both arms and legs motor imagery), and tactile feedback^26^ for 28 months and 34 months respectively. The two patients, referred to here as P1 and P2, were previously referred to as P5 and P6 by Donati *et al*.^14^ and Shokur *et al*.^20,26^.

### Study approval

The study was approved by both the local ethics committee (Associação de Assistência à Criança Deficiente, São Paulo, São Paulo, Brazil #364.027) and the Brazilian federal government ethics committee (CONEP, CAAE: 13165913.1.0000.0085). The experiments were carried out as a feasibility study that did not meet the clinicaltrials.gov definition of an Applicable Clinical Trial. All research activities were carried out in accordance with the guidelines and regulations of the Associação de Assistência à Criança Deficiente and CONEP. The participants signed a written informed consent before enrolling in the study, and also signed a written informed consent for open access publication (print and digital) of their images. The experiments were carried out at the Associação Alberto Santos Dumont para Apoio à Pesquisa (AASDAP), São Paulo.

#### sFES conditioning

An initial screening, which tested all muscles individually, helped set the stimulation amplitude and frequency parameters for the conditioning. The pulse width was fixed at 300 μs for the whole program^65^. The conditioning phase aimed at preparing the muscles progressively to produce a muscle contraction that allowed lower-limb movements of flexion, extension, and abduction, with the final goal to produce a sFES-generated gait. The stimulation time for each muscle increased gradually, up to 40 minutes maximum. The ramp up/down stimulation time decreased from one session to the next one. During the first eight sessions, the patient was maintained in the supine position, and the legs were positioned on rollers to allow free movement. The muscles were first stimulated individually, then in pairs and finally alternating between flexors and extensors (leg-by-leg pattern). The leg-by-leg pattern was applied in two phases: extensors of one leg were stimulated, while the flexors of the opposite leg contracted for 10–15 seconds. The muscles then rested for 2 seconds before the opposite pattern was applied. During the session, a physiatrist supervising the experiment evaluated the muscle response (visual evaluation of the range of motion of the joints and presence or absence of fatigue), the tonus behavior, the sensory perception of the stimulation and the ability to voluntarily improve the muscle contraction together with the stimulation.

For the last session, the patient was supported in an upright position in a body weight support system (Zero-G, Aretech LLC., Ashburn, VA). The stimulation sequence, called stationary gait, differed from the leg-by-leg sequence due to the continuity of the stimulation to extend both lower-limbs. The following criteria were expected for a patient to continue with the FC phase in standing position: adequate muscle responses to allow the hip and knee extension, stable blood pressure, no signs of fatigue during the first 25 minutes, and no spasticity disturbing the stimulation pattern.

#### Body-weight support system

A robotic body weight support system (Zero-G, Aretech LLC., Ashburn, VA) was used to support 65–70% of the weight of the patient during gait. This percentage was selected to provide the minimum suspension required to avoid a crouching position of the lower-limbs, ensuring good contact between the foot and the ground and reducing muscle effort to help avoid premature fatigue.

#### Real-time tracking system

During all experiments, hip and knee joint angles were measured in real-time at 148 Hz. The system used four inertial measurement units (IMU, Trigno TM Inertial Measurement Sensors, Delsys, USA). One sensor was placed on each femur (thigh) and tibia (lower leg) of the patient. The joint angle was computed based on the difference in orientation of the sensors on the adjacent segments^66^. The patient was asked to keep the trunk position straight throughout the training. At the first session, the sensors were positioned on the body relative to anatomical references (patella, tibial tuberosity, and malleolus) and we calculated the parameters needed for the algorithm.

#### Description of the gait pattern and rationale

To produce the gait movement, we designed an activation sequence based on 16 lower-limb muscles stimulated non-invasively. The stimulation pattern, shown in Fig. 1G was determined based on eight gait sub-phases, namely (1) initial contact, (2) load response, (3) mid-stance, (4) terminal stance, (5) pre-swing, (6) initial swing, (7) mid-swing and (8) terminal swing. The stride duration could vary from 4 to 20 seconds. The highest joint range of motion of the human gait takes place in the sagittal plane where flexion and extension of the hip, knee, and ankle occur. We selected extensors and flexors for each joint.

We describe here each muscle activation of the stimulation pattern, considering the start of the stance as the start of the gait cycle. In this position, during initial contact, the vertical reaction force is acting in front of the hip joint promoting an internal flexor moment that will induce hip flexion. The GMx is therefore activated to challenge this internal moment to extend the hip and to align the trunk and pelvis in the orthostatic posture. During the single support phase (that includes mid-stance and terminal stance sub-phases), the patients’ lack of lateral pelvic stabilization could potentially compromise hip joint integrity. To avoid this issue, the Gmd muscle was stimulated to stabilize the pelvis segment in the frontal plane. The Sl muscle was recruited to control the advancement of the tibia in mid-stance, together with the knee extensor, VL, to maintain dynamic stability and extension of the knee at stance.

The pre-swing phase started when the opposite foot touched the ground. At this instant, the Gs was stimulated to assist the knee flexion, and the Hs stimulation reinforced this movement. This condition promoted lifting the heel off the ground. After this, during the initial swing, the RFP was stimulated to flex the hip to lift the foot from the ground. Then the RFP acted to counteract the external lower-limb extension moment promoted by gravity. The RFP stimulation during terminal swing also allowed better control of the downward movement of the limb towards the ground, preparing the limb for the next cycle (next initial contact).

The Hs stimulation was progressively replaced by the VL to extend the knee gradually in the mid- and terminal swing. The TA stimulation promoted ankle dorsiflexion to keep it in a neutral position during the swing phase and to avoid dragging the foot on the ground. The TA stimulation decreased progressively, slightly before the foot touched the ground, to let the foot set down gently on the floor in a flat position, minimizing joint injuries. Three types of different speeds ramps were used (slow, middle, rapid).

#### sFES Stimulator

We used a custom 20-channel stimulator for this project^39,67^. The stimulator sends charge-balanced, rectangular, biphasic pulses. Surface electrodes were used to stimulate 16 selected muscles as shown in Fig. 1C. Two sizes of electrodes were used: 5 × 10 cm (for GMx, Gmd, VL, Hs, Gs) and 5 × 5 cm to target important muscles more locally (RFP) or smaller muscles (TA, Sl). The stimulator allowed the modulation of pulse width, frequency and current intensity at a refresh rate of 10 ms. The pulse width was maintained at 300 μs as this was previously found as an optimal value^65^. By default, a frequency of 30 Hz was used^68^. This electrostimulator and the risk analysis associated report has been approved by the SwissMedic Ethical Committee for Clinical Investigations (SwissMedic Ethical approval, Ref MD-2007-MD-0031).

#### PI controller

The PI controller ran at 50 Hz and adapted the stimulation amplitude to reach the predefined target angles (see Description of the gait pattern and rationale). For safety, the maximum current modulation was saturated within a range of 20% above or below the command. The parameters for the PI controllers were found using the pure PI control (i.e., without pre-established stimulation pattern) on healthy subjects to achieve typical joint trajectories (ramp target, sinusoidal target and kinematics curves from a normal gait cycle). Proportional and integral gain, Kp and Ki were respectively set to 0.5 and 0.001.

#### PI controller characterization experiment

To evaluate the effect of the PI controller, we designed a specific experiment that isolated only the knee extension. The patient was sitting in his wheelchair with his feet hanging. The vastus of each leg was stimulated alternately to induce left and right knee extension. We designed the desired angle trajectory to be composed of ramps and plateaus to study the characteristics of the controller with constant and changing targets. We pseudo-randomized the trials where the PI controller was on (PI-on) and trials where it was off (PI-off) (three PI-off and three PI-on were shuffled every six repetitions). Only the vastus muscle was stimulated to act as knee extensor, and the knee flexion was performed passively by gravity. The whole experiment was composed of 100 repetitions (50 open-loop, PI-off, and 50 closed-loop, PI-on). The current amplitude model for the open-loop was composed of ramps and plateaus between two amplitudes. Those two amplitudes were selected during preliminary tests on the same day to obtain two levels of extension close to the desired target.

#### Custom visualization system and LEDs

During the B + FL phase, two arrays of four LEDs were placed on each side of the walker to indicate the output of the EEG classifier and the experiment’s protocol. The left and rightmost LEDs informed the patients of the experiment with a simple color convention: black (turned off) for idle time, pink for focus, and left or right blue LED for corresponding leg motor imagery. At the end of each trial, the patient could see if he succeeded or failed the trial (green or red LED). Patients’ EEG classifier value was discretized in the [−1.5; +1.5] range and shown using the six remaining LEDs.

#### EEG and BMI

We used the opensource software OpenViBE 0.16.0, for the acquisition and processing of EEG signals^69^. EEG signals were acquired at 2000 Hz with the V-amp amplifier (Brain Products, GmbH). Sixteen channels were recorded and clustered around the leg representation of the primary sensorimotor cortex (Fig. 1E). We used actiCAP active electrodes, connected to the wireless MOVE (both Brain Products, GmbH), carried with the patient. The wireless receiver was directly connected to the amplifier via USB to a computer. EEG signals were band-pass filtered at 8–30 Hz (mu and beta), corresponding to the well-documented frequency band for motor-related EEG activity^70^.

We then used two algorithms for the detection of motor imagery: the Common Spatial Patterns (CSP) algorithm^46^ as a spatial filter and Linear Discriminant Analysis (LDA) for classification. The CSP aims at learning spatial filters, such that the variance of the signals is maximized for one class (e.g., one mental imagery task) and minimized for the other class. The Linear Discriminant Analysis (LDA) algorithm was used to estimate a linear hyperplane to separate feature vectors from two classes)^71^. In our case, if the LDA output was positive, the feature vector was assigned to the right motor imagery class. Otherwise, it was assigned to the left leg motor imagery class. Every BMI session started with 8 minutes of training (40 trials, 20 left and right leg motor imageries, randomized order). Each trial consisted of 1-second preparation, followed by 1.25 s of cue presentation and a 3.75 s motor imagery period. The intertrial time was randomly chosen between 1.5 and 3.5 seconds.

Patients were instructed to produce motor imagery involving only one leg at the time as, e.g., ‘imagine kicking a ball with your right leg,’ ‘imagine making a circular movement with your left ankle,’ and to avoid motor imageries that involved the movement of both legs such as ‘cycling’ or ‘walking.’ We used a 5-fold, cross-validation for calculation of the classifier accuracy.

During this acquisition, the patient already had the sFES electrodes placed and connected to the stimulator (with the stimulator turned off); he was standing, in the BWS system, but without suspension and was supported in an upright position. Before the first BMI run, the patient remained for 30 seconds without motor imagery in a standing position to record the baseline classifier. The mean of this signal was subtracted from the classifier for the run to avoid any offset. The baseline could be calculated one more time before the fourth run if needed to compensate for any new offset.

During the online control, we used the parameters for the CSP and the LDA that were calculated during the training phase. The only difference with the training phase was a threshold of 0.1 on the classifier value to reduce chances of false positives: we considered detection of left or right motor imagery only when the classifier was, respectively, smaller than −0.1 or above 0.1.

#### Tactile shirt

The tactile shirt was composed of the three vibrators aligned on the ulna as described in^26^. Patients received continuous tactile feedback going from the wrist to the elbow (apparent movement as described in^45^) coinciding with the stimulation of the lower-limb muscles to indicate the beginning the swing (start of phase six, Fig. 1G). At the end of phase eight, they received stimulation of all three vibrators together for 600 ms.

#### Calculation of the chance level for the BMI

The baseline data was gathered during resting time with the same patients, collected at the onset of each BMI session. All experimental conditions were the same as those for the current experimental paradigm. Patients were using the same EEG cap, and setup (standing position). EEG signals were recorded for 30 seconds prior to the session. We gathered all the baseline datasets and cut them into 4 s time windows corresponding to step trials. Then we applied the exact same algorithm and simulated a sequence of runs based on these datasets. We report the statistics for chance level, obtained by simulating 600 steps (50 runs of 12 steps): 5.72 + − 1.82 (mean + −SD) correct steps performed over 12 steps run, and the time to perform a step is on average 3.31 + −0.84[s].

#### Session interruption and data exclusion

A clinical check was done before and during the sessions. If the patient presented abnormal low blood pressure or severe cases of spasticity before or during the session, the session was, respectively, not initiated or interrupted.

#### Qualitative visual gait evaluation

Each evaluation session had six FL runs; each run had 12 steps. Step time was pseudo-randomized per run (8 s/step, 6 s/step or 4 s/step). Video clips of the patients’ runs were cut, shuffled and given to an expert physiotherapist blinded to the conditions of the experience, with a questionnaire developed by our group (referred to here as visual gait score for sFES, see Supplementary Table S4 for details). The questionnaire included three control, five high-level and seven kinematics-oriented questions. The answers were Likert-type, ranging from 1 to 5. The control questions assessed respectively whether the patient exhibited spasticity, clear signs of fatigue or reduction of muscle response to the stimulation during the run. If any of the three control questions had a score superior or equal to 4, the run was discarded. No runs were removed for any of the two sessions used for the tactile-on versus tactile-off experiment with patient P1. One of the runs of patient P2 and none of P1 were discarded from the qualitative analysis of sFES-generated walk.

#### 3D Gait analysis

Standard gait-analysis procedures were employed to collect data using a motion-capture system comprised of 12 cameras and a 4 Mb resolution (Raptor-4) and the Cortex 6.0 software (Motion Analysis, Santa Rosa, CA, USA). The kinematic data were acquired at 150 Hz. The marker-set protocol adopted was comprised of 28 anatomical reflective markers (Helen Hayes marker set). Markers were placed directly onto the skin in the patient’s pelvic and lower-extremity segments. Offline, raw marker-trajectory data were filtered using a 4th-order, low-pass Butterworth filter with a cut-off frequency of 10 Hz. Data processing was performed using the Cortex software version 6.0. Visual 3D software (C-motion Inc., Germantown, MD, USA) was used to perform all kinematics calculations. The foot strike and toe-off were determined when the velocity of the heel and toe markers respectively crossed a 20 mm/s threshold level. The definition of the anatomical-segment coordinate system was used to determine the 3D position and orientation of the lower extremity and pelvis segments through a combination of anatomical-frame conventions proposed previously^72^. Patient P2 performed 54 full gait cycles, with 101 time-normalized points, which corresponded to the time-normalized angles (pelvis, hip, knee, ankle, and foot).

## Supplementary information

Supplementary Info

Supplementary Video 1

Supplementary Video 2

Supplementary Video 3

Supplementary Video 4

## Data Availability

The custom code used for the experiments and the data that support the findings of this study are available from the corresponding author upon reasonable request.
