# Author Correction: Non-invasive, Brain-controlled Functional Electrical Stimulation for Locomotion Rehabilitation in Individuals with Paraplegia

**DOI:** 10.1038/s41598-019-54834-3

**Published:** 2019-12-04

**Authors:** Aurelie Selfslagh, Solaiman Shokur, Debora S. F. Campos, Ana R. C. Donati, Sabrina Almeida, Seidi Y. Yamauti, Daniel B. Coelho, Mohamed Bouri, Miguel A. L. Nicolelis

**Affiliations:** 1 Neurorehabilitation Laboratory, Associação Alberto Santos Dumont para Apoio à Pesquisa (AASDAP), São Paulo, 05440-000 Brazil; 2 STI IMT, École Polytechnique Fédérale de Lausanne, Lausanne, Switzerland; 3 Associação de Assistência à Criança Deficiente (AACD), São Paulo, 04027-000 Brazil; 4 Biomedical Engineering, Federal University of ABC, São Bernardo do Campo, SP 09606-045 Brazil; 5 Department of Neurobiology, Duke University Medical Center, Durham, NC 27710 USA; 6 Duke Center for Neuroengineering, Duke University, Durham, NC 27710 USA; 7 Department of Biomedical Engineering, Duke University, Durham, NC 27708 USA; 8 Department of Neurology, Duke University, Durham, NC 27710 USA; 9 Department of Neurosurgery, Duke University, Durham, NC 27710 USA; 10 Department of Psychology and Neuroscience, Duke University, Durham, NC 27708 USA; 11 Edmond and Lily Safra International Institute of Neuroscience, Macaíba, Brazil

Correction to: *Scientific Reports* 10.1038/s41598-019-43041-9, published online 01 May 2019

This Article contains typographical errors in the Acknowledgements section.

“This study was funded by the Brazilian Financing Agency for Studies and Projects (FINEP 01·12·0514·00), Brazilian Ministry of Science, Technology, and Innovation (MCTI). We acknowledge the National Institute of Science and Technology (INCT) Brain Machine-Interface (INCEMAQ) CNPq 573966/2008-7 of Brazilian Ministry of Science, Technology, and Innovation (MCTI/CNPq/FNDCT/CAPES/FAPERN). We want to thank Neiva Paraschiva, Adriana Ragoni, Andrea Arashiro, Maria Cristina Boscarato, Fabio Asnis, Nathan Rios, Dr. Tiago Kunrath, and Susan Halkiotis (Duke University) for their work, help, and support for this study. We finally want to thank the patients for their long-term commitment, and their trust in this research and our team.”

should read:

“This study was funded by the Brazilian Financing Agency for Studies and Projects (FINEP 01·12·0514·00), Brazilian Ministry of Science, Technology, Innovation, and Communication (MCTIC). We acknowledge the National Institute of Science and Technology (INCT) Brain Machine-Interface (INCEMAQ, Siconv 704134/2009) of the National Council for Scientific and Technological Development (CNPq), Brazilian Ministry of Science, Technology, Innovation and Communication (MCTIC). Maria Cristina Boscarato, Fabio Asnis, Nathan Rios, Dr. Tiago Kunrath, and Susan Halkiotis (Duke University) for their work, help, and support for this study. We finally want to thank the patients for their long-term commitment, and their trust in this research and our team.”

