# Supplementary material for: Non-invasive, Brain-controlled Functional Electrical Stimulation for Locomotion Rehabilitation in Individuals with Paraplegia
Source: Sci Rep. 2019 May 1;9:6782. doi: 10.1038/s41598-019-43041-9 (PMC6494802; doi:10.1038/s41598-019-43041-9)
Supplement: Supplementary file 1 — Supplementary Info [file 41598_2019_43041_MOESM1_ESM.docx]

**Supplementary Materials**

Title: **Non-invasive, Brain-controlled functional electrical stimulation for locomotion rehabilitation in individuals with** **PARAPLEGIA**

**Authors**: Aurelie Selfslagh ^1,2,*^, Solaiman Shokur ^1,^**^*^**, Debora S.F. Campos^1^, Ana R. C. Donati^1,3^, Sabrina Almeida^1,3^, Seidi Y. Yamauti^1^, Daniel B. Coelho^4^, Mohamed Bouri^2^, Miguel A. L. Nicolelis^1, 5,6,7,8,9,10,11^

* AS and SSH have equally contributed to this work

**Affiliations:**

- ^1^ Neurorehabilitation Laboratory, Associação Alberto Santos Dumont para Apoio à Pesquisa (AASDAP), São Paulo, Brazil, 05440-000;
- ^2^ STI IMT, École Polytechnique Fédérale de Lausanne, Lausanne, Switzerland
- ^3^ Associação de Assistência à Criança Deficiente (AACD), São Paulo, Brazil, 04027-000;
- ^4^ Biomedical Engineering, Federal University of ABC, São Bernardo do Campo, SP, Brazil. 09606-045.
- ^5^ Department of Neurobiology, Duke University Medical Center, Durham, NC, 27710;
- ^6^ Duke Center for Neuroengineering, Duke University, Durham, NC, 27710;
- ^7^ Department of Biomedical Engineering, Duke University, Durham, NC, 27708;
- ^8^ Department of Neurology, Duke University, Durham, NC, 27710;
- ^9^ Department of Neurosurgery, Duke University, Durham, NC, 27710;
- ^10^ Department of Psychology and Neuroscience, Duke University, Durham, NC, 27708;
- ^11^ Edmond and Lily Safra International Institute of Neurosciences of Natal, Natal, Brazil.


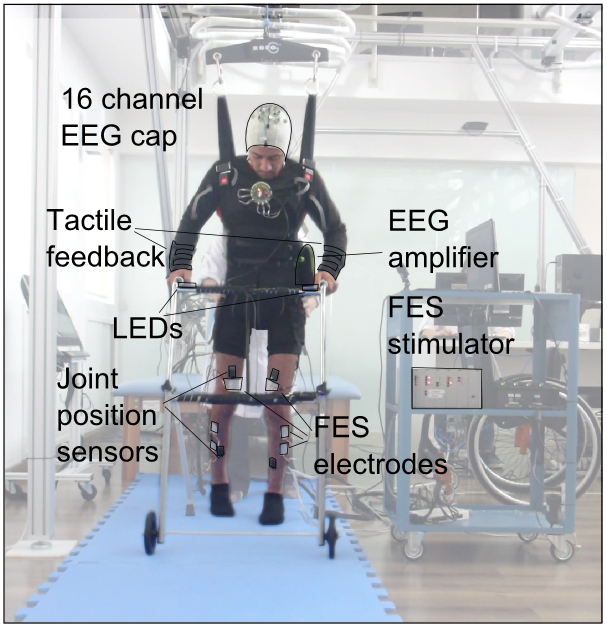


**Figure S1.** Patient with the 16 channel EEG system, a portable EEG amplifier, the wearable haptic device, the 16 sFES electrodes (only four pairs visible in this image), the IMU sensors and the walker. An array of LEDs integrated with the walker gave online feedback on patients’ brain classifier as well as the current trial’s state. The 16 Channel sFES stimulator system was mounted on a moving cart.


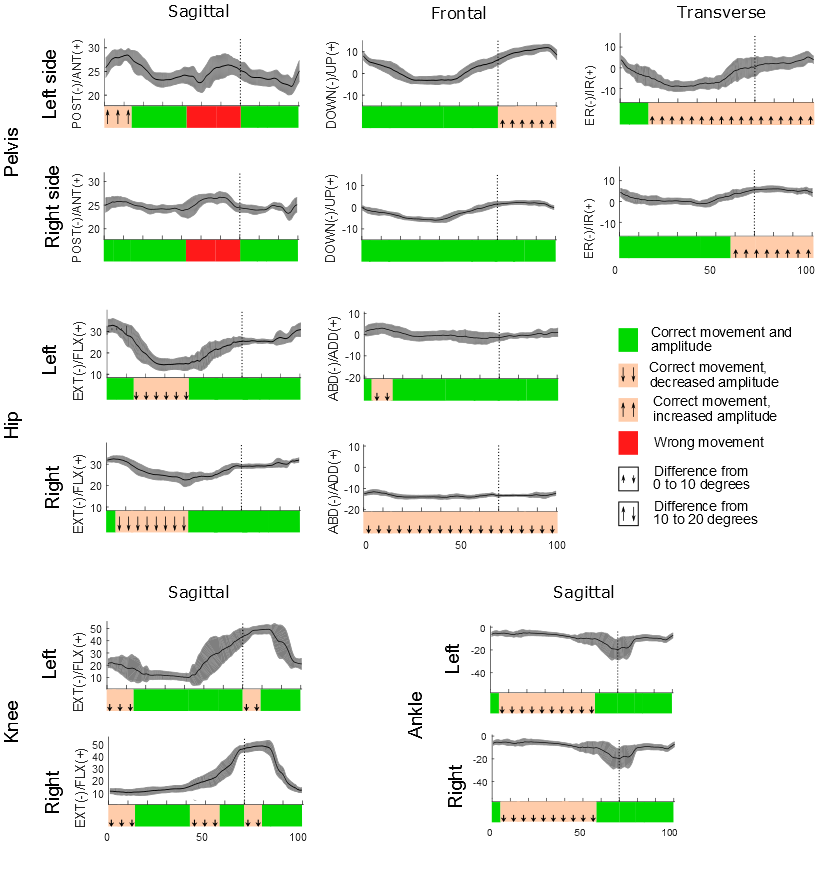


**Figure S2.** In a physiological walk ^1^, the pelvis segment, hip, knee, and ankle joints describe a sequence of pre-defined movements on the sagittal plane (flexion-extension), the frontal plane (adduction-abduction) and the transversal plane (internal rotation-external rotation). The greater range of motion occurs in the sagittal plane and is responsible for the displacement of the body during the gait. The movements of the joints on the adduction-abduction axis and rotation axis are smaller and necessary for biomechanical efficiency, for example helping with shock absorption. The analysis was made on 54 full steps recorded from patient P2. During this session the tactile shirt was turned off and the control was in automatic mode (without BMI). We report for both right and left legs, in green, the periods of the gait where the recorded movements followed the expected angles; in orange the periods where the movement was correct but with either a higher amplitude (arrows pointing up) or lower (arrows pointing down) than expected; and in red when the movement was wrong. The kinematics of the pelvis segment on the sagittal plane shows a small increase in the average anteversion in the cycle, which could be due to hypotonia of the abdominal muscles. Also, the range of motion is above the expected angle. We attribute this to the patient's tendency to exaggerate the leaning of the trunk, in order to facilitate the contralateral swing. During the initial contact and load response, there is an adequate increase of the anteversion. However, the amplitude is higher than expected (30 degrees instead of 15). The pelvis remains correctly stable (without anterior-posterior movement) at the mid stance. We observed an inappropriate movement in the terminal stance and anteversion movement of the pre-swing. They were due to, respectively, an exaggerated movement during the terminal swing and the initial contact/load response of the contralateral leg, which were again induced by an exaggerated movement of the patient’s trunk. Finally, the observed movement during the whole swing phase was adequate.


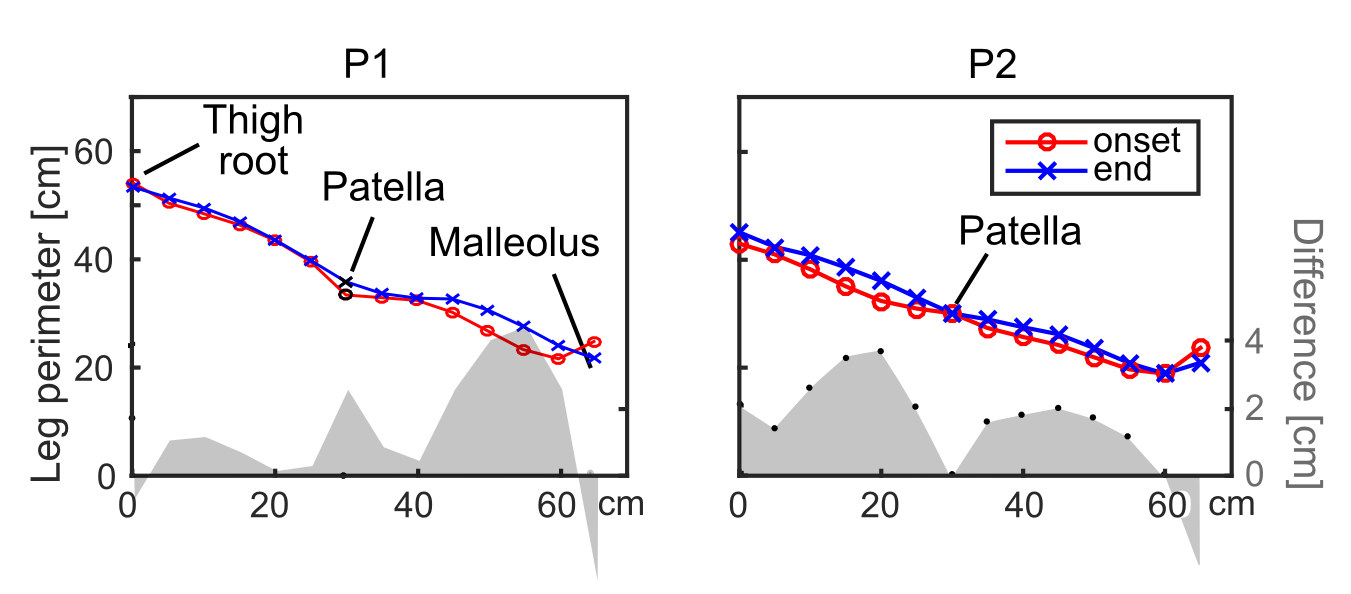


**Figure S3.** Perimeter at the onset and end of the BFRN protocol and the difference in the two assessments (gray shaded graph).

**Supplementary Tables**

**Table S1.** ISNCSCI assessment for patient P1 done at the onset of the WANR ^2^ (M1), at the onset of the protocol described in the current work (M2) and at the end of the protocol (M5). LT: Light Touch; PP: Pin Prick; DAP: Deep anal pressure; VAC: Voluntary anal contraction; UER/UEL: Upper Extremity Right/Left; LER/LEL: Lower Extremity Right/Left; UEMS/LEMS: Upper/Lower Extremity Motor Score; ZPP: Zone of Partial Preservation.

|  |  | **M1** | | **M2** | | **M5** | |
| --- | --- | --- | --- | --- | --- | --- | --- |
|  |  | **Right** | **Left** | **Right** | **Left** | **Right** | **Left** |
| LT | S4-S5 | 0 | 0 | 0 | 0 | 0 | 0 |
|  | **RLT + LLT** | **29** | **29** | **34** | **34** | **34** | **32** |
|  | **LT TOTAL** | **58** | | **68** | | **66** | |
| **PP Prick** | S4-S5 | 0 | 0 | 1 | 1 | 1 | 1 |
|  | **RPP + LPP** | **29** | **29** | **32** | **32** | **32** | **31** |
|  | **PP TOTAL** | **58** | | **64** | | **63** | |
|  | **DAP** | No | | Yes | | Yes | |
|  | **VAC** | No | | No | | No | |
|  |  |  |  |  |  |  |  |
| **Up. extr.** | **C5 - Elbow flexors** | 5 | 5 | 5 | 5 | 5 | 5 |
|  | **C6 - Wrist extensors** | 5 | 5 | 5 | 5 | 5 | 5 |
|  | **C7 - Elbow extensors** | 5 | 5 | 5 | 5 | 5 | 5 |
|  | **C8 - Finger flexors** | 5 | 5 | 5 | 5 | 5 | 5 |
|  | **T1 - Finger abductors** | 5 | 5 | 5 | 5 | 5 | 5 |
|  |  |  |  |  |  |  |  |
| **Lower. extr.** | **L2 - Hip flexors** | 0 | 0 | 1 | 1 | 1 | 1 |
|  | **L3 - Knee extensors** | 0 | 0 | 1 | 1 | 1 | 2 |
|  | **L4 - Ankle dorsiflexors** | 0 | 0 | 0 | 0 | 0 | 0 |
|  | **L5 - Long toe extensors** | 0 | 0 | 0 | 0 | 0 | 0 |
|  | **S1 - Ankle plantar flexors** | 0 | 0 | 0 | 0 | 1 | 1 |
|  |  |  |  |  |  |  |  |
|  | **UER - UEL** | **25** | **25** | **25** | **25** | **25** | **25** |
|  | **UEMS TOTAL** | **50** | | **50** | | **50** | |
|  | **LER - LEL** | **0** | **0** | **2** | **2** | **3** | **4** |
|  | **LEMS TOTAL** | **0** | | **4** | | **7** | |
|  |  |  |  |  |  |  |  |
| **Neuro. Levels** | **Sensory** | T7 | T7 | T8 | T8 | T8 | T8 |
|  | **Motor** | T7 | T7 | T8 | T8 | T8 | T8 |
|  | **Neurological Level of Injury** | T7 | | T8 | | T8 | |
|  | **Complete or Incomplete** | C | | I | | I | |
|  | **ASIA Impairment Scale** | A | | C | | C | |
|  | **ZPP Sensory** | T8 | T8 |  |  |  |  |
|  | **ZPP Motor** | T7 | T7 |  |  |  |  |
|  | **Comments** |  | | Sensory function at S4-S5 and motor function present more than 3 levels (L3) below the motor level (T8) | | Sensory function at S4-S5 and motor function present more than 3 levels (S1) below the motor level (T8) | |

**Table S2.** ISNCSCI assessment for patient P2 done at the onset of the WANR ^2^ (M1), at the onset of the protocol described in the current work (M4) and at the end of the protocol (M5). LT: Light Touch; PP: Pin Prick; DAP: Deep anal pressure; VAC: Voluntary anal contraction; UER/UEL: Upper Extremity Right/Left; LER/LEL: Lower Extremity Right/Left; UEMS/LEMS: Upper/Lower Extremity Motor Score; ZPP: Zone of Partial Preservation.

|  |  | **M1** | | **M4** | | **M5** | |
| --- | --- | --- | --- | --- | --- | --- | --- |
|  |  | **Right** | **Left** | **Right** | **Left** | **Right** | **Left** |
| LT | S4-S5 | 0 | 0 | 0 | 0 | 1 | 1 |
|  | **RLT + LLT** | 24 | 23 | 29 | 29 | 32 | 33 |
|  | **LT TOTAL** | **47** | | **58** | | **65** | |
| **PP Prick** | S4-S5 | 0 | 0 | 0 | 0 | 1 | 1 |
|  | **RPP + LPP** | 24 | 23 | 30 | 27 | 33 | 32 |
|  | **PP TOTAL** | **47** | | **57** | | **65** | |
|  | **DAP** | No | | Yes | | Yes | |
|  | **VAC** | No | | No | | No | |
|  |  |  |  |  |  |  |  |
| **Up. extr.** | **C5 - Elbow flexors** | 5 | 5 | 5 | 5 | 5 | 5 |
|  | **C6 - Wrist extensors** | 5 | 5 | 5 | 5 | 5 | 5 |
|  | **C7 - Elbow extensors** | 5 | 5 | 5 | 5 | 5 | 5 |
|  | **C8 - Finger flexors** | 5 | 5 | 5 | 5 | 5 | 5 |
|  | **T1 - Finger abductors** | 5 | 5 | 5 | 5 | 5 | 5 |
|  |  |  |  |  |  |  |  |
| **Lower. extr.** | **L2 - Hip flexors** | 0 | 0 | 0 | 0 | 1 | 1 |
|  | **L3 - Knee extensors** | 0 | 0 | 1 | 1 | 2 | 2 |
|  | **L4 - Ankle dorsiflexors** | 0 | 0 | 0 | 0 | 1 | 1 |
|  | **L5 - Long toe extensors** | 0 | 0 | 0 | 0 | 0 | 1 |
|  | **S1 - Ankle plantar flexors** | 0 | 0 | 0 | 0 | 1 | 1 |
|  |  |  |  |  |  |  |  |
|  | **UER - UEL** | **25** | **25** | **25** | **25** | **25** | **25** |
|  | **UEMS TOTAL** | **50** | | **50** | | **50** | |
|  | **LER - LEL** | **0** | **0** | **1** | **1** | **5** | **6** |
|  | **LEMS TOTAL** | **0** | | **2** | | **11** | |
|  |  |  |  |  |  |  |  |
| **Neuro. Levels** | **Sensory** | T4 | T4 | T5 | T5 | T5 | T5 |
|  | **Motor** | T4 | T4 | T5 | T5 | T5 | T5 |
|  | **Neurological Level of Injury** | T4 | | T5 | | T5 | |
|  | **Complete or Incomplete** | C | | I | | I | |
|  | **ASIA Impairment Scale** | A | | C | | C | |
|  | **ZPP Sensory** | T6 | T5 |  |  |  |  |
|  | **ZPP Motor** | T4 | T4 |  |  |  |  |
|  | **Comments** |  | | Sensory function at S4-S5 and motor function present more than 3 levels (L3) below motor level (T5) | | Sensory function at S4-S5 and motor function present more than 3 levels (S1) below motor level (T5) | |

**Table S3. Patients’ demography.** 1) Neurological level of injury is calculated with the ISNCSCI assessment. 2) Anatomic lesion level (MRI guided). 3) Walk again neurorehabilitation. 4) BMI, FES Neurorehabilitation.

|  | **Neurological level of injury ^1^** | | **Anatomical lesion level^2^** | **AIS onset WANR^3^** | **AIS end of WANR^3^** | **AIS onset BFNR^4^** | **Time since lesion** |
| --- | --- | --- | --- | --- | --- | --- | --- |
|  | **R** | **L** |  |  |  |  |  |
| **P1** | T7 | T7 | T7-T10 | A | C | C | 4.5 years |
| **P2** | T4 | T4 | T3-T5 | A | C | C | 10 years |

**Table S4: Questionnaire used for the visual gait score for sFES analysis**. All Responses were a Likert-type; TD-TA: totally disagree, disagree, neither agree nor disagree, agree, totally agree; NV-AL: never, rarely, every once in a while, sometimes, almost always.

| **1. Body position behavior** | Response type |
| --- | --- |
| A) Trunk alignment was adequate. | TD-TA |
| B) The patient used upper-limb compensatory movements. | TD-TA |
| C) Coordination between walking progression and walker movement was adequate. | TD-TA |
| D) The patient was looking at her/his own body. | NV-AL |
| E) Verbal instructions: therapist gave instructions to the patient during the run to perform gait correctly. | NV-AL |
| **2. Expected gait events: stance + swing phases** |  |
| **Stance phase** |  |
| A) The posture of the foot during the initial contact was plantigrade. | NV-AL |
| B) The posture of the foot during the single support was plantigrade. | NV-AL |
| C) The knee position during the single support maintained a 0-10 º range. | NV-AL |
| **Swing phase** | NV-AL |
| A) The foot was dragging on the floor during the initial swing. | NV-AL |
| B) The knee flexion during the swing phase maintained the 55-65 degrees range. | NV-AL |
| C) The ankle dorsiflexion during the swing maintained at the neutral position. | NV-AL |
| **3. Control questions** | NV-AL |
| A) The muscle responses were reduced during the gait. | NV-AL |
| B) The patient exhibited spasticity. | NV-AL |
| C) Patient lower-limbs exhibited signs of fatigue. | NV-AL |

**Supplementary Movie Legends**

**Movie S1:** 3D gait reconstruction for sFES-generated gait test with patient P2.

**Movie S2:** Patient P1 during B+FL run at the end of the protocol. He controlled successfully six steps of 11. Permission for publication granted by Associação Alberto Santos Dumont para Apoio à Pesquisa (AASDAP), São Paulo, Brazil.

**Movie S3:** Patient P2 during B+FL run at the end of the protocol. He controlled successfully all 11 steps shown. Permission for publication granted by Associação Alberto Santos Dumont para Apoio à Pesquisa (AASDAP), São Paulo, Brazil.

**Movie S4:** Patient P2 clinical motor evaluations. Permission for publication granted by Associação Alberto Santos Dumont para Apoio à Pesquisa (AASDAP), São Paulo, Brazil.

**Bibliography**

1. Charalambous, C. P. Repeatability of kinematic, kinetic, and electromyographic data in normal adult gait. in *Classic Papers in Orthopaedics* 399–401 (2014). doi:10.1007/978-1-4471-5451-8_101

2. Donati, A. R. C. *et al.* Long-Term Training with a Brain-Machine Interface-Based Gait Protocol Induces Partial Neurological Recovery in Paraplegic Patients. *Sci. Rep.* **6,** 30383 (2016).
